# Effects of an anti-inflammatory VAP-1/SSAO inhibitor, PXS-4728A, on pulmonary neutrophil migration

**DOI:** 10.1186/s12931-015-0200-z

**Published:** 2015-03-20

**Authors:** Heidi C Schilter, Adam Collison, Remo C Russo, Jonathan S Foot, Tin T Yow, Angelica T Vieira, Livia D Tavares, Joerg Mattes, Mauro M Teixeira, Wolfgang Jarolimek

**Affiliations:** Drug Discovery Department, Pharmaxis Ltd, 20 Rodborough Road, Frenchs Forest, Sydney, NSW 2086 Australia; The University of Newcastle & Vaccines, Infection, Viruses & Asthma, Newcastle, Australia; Laboratório de Imunologia e Mecânica Pulmonar, Departamento de Fisiologia e Biofísica, Universidade Federal de Minas Gerais, Av. Antonio Carlos, 6627, Pampulha, 31270-901 Belo Horizonte, MG Brazil; Laboratório de Imunofarmacologia, Departamento de Bioquímica e Imunologia, Universidade Federal de Minas Gerais, Av. Antonio Carlos, 6627, Pampulha, 31270-901 Belo Horizonte, MG Brazil; School of Medical & Molecular Biosciences, University of Technology Sydney, City Campus, PO Box 123 Broadway, 2007 Sydney, NSW Australia

**Keywords:** Neutrophils, Adhesion molecules, VAP-1/SSAO, Lung inflammation, COPD

## Abstract

**Background and purpose:**

The persistent influx of neutrophils into the lung and subsequent tissue damage are characteristics of COPD, cystic fibrosis and acute lung inflammation. VAP-1/SSAO is an endothelial bound adhesion molecule with amine oxidase activity that is reported to be involved in neutrophil egress from the microvasculature during inflammation. This study explored the role of VAP-1/SSAO in neutrophilic lung mediated diseases and examined the therapeutic potential of the selective inhibitor PXS-4728A.

**Methods:**

Mice treated with PXS-4728A underwent intra-vital microscopy visualization of the cremaster muscle upon CXCL1/KC stimulation. LPS inflammation, *Klebsiella pneumoniae* infection, cecal ligation and puncture as well as rhinovirus exacerbated asthma models were also assessed using PXS-4728A.

**Results:**

Selective VAP-1/SSAO inhibition by PXS-4728A diminished leukocyte rolling and adherence induced by CXCL1/KC. Inhibition of VAP-1/SSAO also dampened the migration of neutrophils to the lungs in response to LPS, *Klebsiella pneumoniae* lung infection and CLP induced sepsis; whilst still allowing for normal neutrophil defense function, resulting in increased survival. The functional effects of this inhibition were demonstrated in the RV exacerbated asthma model, with a reduction in cellular infiltrate correlating with a reduction in airways hyperractivity.

**Conclusions and implications:**

This study demonstrates that the endothelial cell ligand VAP-1/SSAO contributes to the migration of neutrophils during acute lung inflammation, pulmonary infection and airway hyperractivity. These results highlight the potential of inhibiting of VAP-1/SSAO enzymatic function, by PXS-4728A, as a novel therapeutic approach in lung diseases that are characterized by neutrophilic pattern of inflammation.

## Background

Several inflammatory diseases of the lung including chronic obstructive pulmonary disease (COPD), cystic fibrosis (CF), acute lung injury and bronchiolitis obliterans syndrome are predominantly characterized by neutrophilic infiltration [1-4]. Neutrophils are polymorphonuclear phagocytic granulocytes and during normal immune responses act as the first line of defense against microorganisms by finding and neutralizing bacteria and fungi [5]. Excessive neutrophilia coupled with inadequate removal of neutrophils (and their byproducts) results in the subsequent and persistent release of a number of inflammatory mediators and proteinases that contribute to progressive fibrosis and destruction of the lung parenchyma [6-8].

Integral to the proper functioning of neutrophils in lung defense is their ability to egress from the microvasculature and migrate through tissues to the targeted site. Upon injury of organs, neutrophils up-regulate expression of cell surface receptors (such as CD44 and CD11b) [9,10]; these subsequently bind to adhesion molecules on endothelial cells (E-selectin, P-selectin, VAP-1/SSAO, ICAM-1), enabling neutrophil transmigration through the endothelial cell lining into the underlying parenchyma [5,11]. In the pulmonary microcirculation however, neutrophil migration primarily occurs from the capillaries, and so far only neutrophil rolling and deformability (not adhesion) have been implicated in this process [12-16].

Given the potential of targeting neutrophil migration via their adhesion process, a number of small molecules have now been developed against neutrophil inflammation. Targeting P-selectin glycoprotein ligand 1 (PSGL-1/CD162) and Mac-1 (CD11b/CD18), not only inhibits pulmonary recruitment of neutrophils but also reduces lung damage in sepsis (29, 30). Neutralization of CD44 reduced cecal ligation puncture (CLP) induced pulmonary accumulation of neutrophils. Studies have also shown that neutrophilic inflammation induced by lipopolysaccharides (LPS) is attenuated in CXCR2 deficient mice and small molecule CXCR2 antagonists inhibit pulmonary neutrophilic inflammation induced by inhaled LPS [17,18]. Administration of the CXCR2 antagonists SCH527123 and SB-656933 has been shown to inhibit the increased neutrophil content in sputum as a result of ozone challenge in normal volunteers, while AZD8309 also was shown to be efficacious during inhaled LPS challenge in normal volunteers [19-21].

Another potential target for impacting neutrophil inflammation is to inhibit their migration via the adhesion molecule VAP-1/SSAO. VAP-1 is a homodimeric 170-kDa sialoglycoprotein that regulates adhesion and consequently transendothelial migration of leukocytes [22]. It is unique amongst the adhesion molecules as it also functions as a semicarbazide-sensitive amine oxidase (SSAO), which is part of a family of amine oxidases capable of oxidizing primary amines (e.g. methylamine) to the corresponding aldehyde (e.g. formaldehyde), releasing hydrogen peroxide and ammonia. It is highly expressed on the endothelium of the lungs and trachea, whereas absent from leukocytes and epithelial cells [23]. Although the exact function of SSAO in the lung is not well elucidated, it has been suggested that SSAO might be involved in the recruitment of inflammatory cells during pulmonary inflammation [23-25].

VAP-1/SSAO overexpressing transgenic animals show higher baseline cell counts in the BALF (bronchoalveolar lavage fluid) than in non-transgenic mice, indicative of increased lung inflammation, due to increased VAP-1/SSAO levels [25]. The pro-inflammatory effect of VAP-1/SSAO was evident when transgenic mice were stimulated with LPS; inflammation was significantly higher in the transgenic than in the non-transgenic mice [25]. Neutrophil migration through the endothelium was shown to be dependent on both the adhesive and the enzymatic functions, with mAb and small molecule enzyme inhibitors reducing the influx of neutrophils. However, these effects were not additive, indicative of a common downstream process [22,24].

The present study demonstrates for the first time *in vivo*, that inhibition of the enzymatic function of VAP-1/SSAO by a small molecule impairs neutrophil migration by mediating tethering and rolling onto the vascular endothelium. Using the small molecule inhibitor PXS-4728A, a crucial role for the enzymatic function of VAP-1/SSAO was identified in the pathogenesis of neutrophil lung inflammation, injury and airway hyperreactivity. This article provides support that targeting VAP-1/SSAO is a potential strategy to inhibit the underlying neutrophil inflammation that contributes to the progression of COPD and other respiratory diseases.

## Materials and methods

### Compound chemical properties

**Figure 1:**
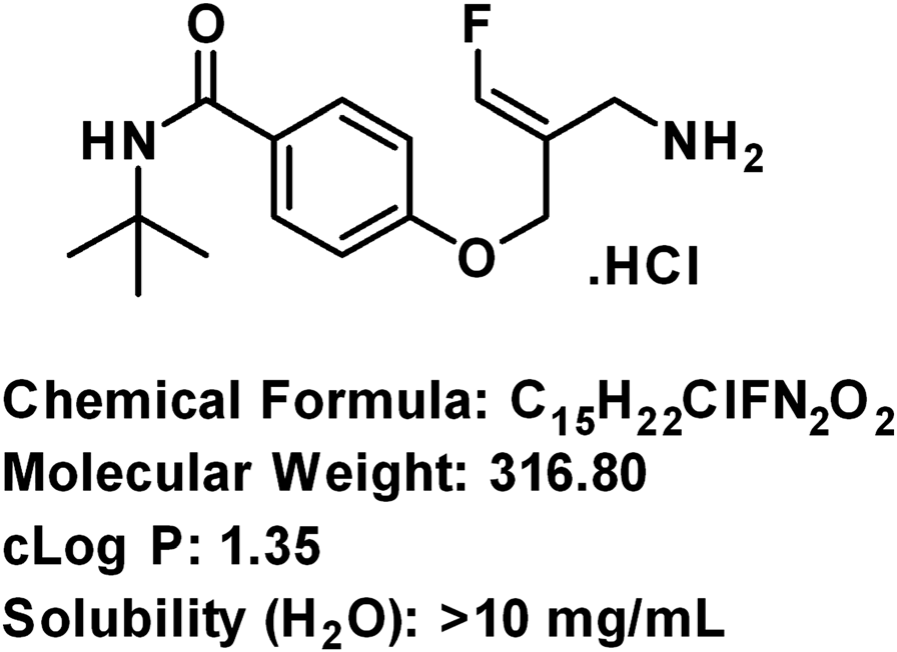
**Structure of PXS-4728A.**

PXS-4728A (Figure 1) is a low molecular weight fluoro-allylamine and chemically similar to the previously described VAP-1/SSAO inhibitor PXS-4681A [24].

### SSAO fluorometric enzyme activity assay

Enzymatic activity measurement was based on the production of H_2_O_2_ as described previously [24]. When measuring activity from tissue, samples were incubated with 0.5 mM of pargyline, to inhibit any potential endogenous monoamine oxidase A and B. In a 384 well plate (Greiner 384 Well Black Microclear Base), 25 μl of the samples were incubated for 30 min at 37°C with or without the specific SSAO inhibitor (1 μM Mofegiline). A reaction mixture containing amplex red (120 μM; Life Technologies, Australia, A12222), horseradish peroxidase (1.5 U/ml; Sigma-Aldrich; P2088-5KU) and Benzylamine (600 μM for rhSSAO; 80 μM for mouse tissue, 30 μM for rat tissue) was prepared in 0.1 M sodium phosphate buffer. 25 μl of the reaction mixture were added into each well after the 30 min incubation. The relative fluorescence units (RFU) were read every 2.5 min for 30 min at 37°C, excitation 565 nm and emission 590 (Optima, BMG labtech), and the slope of the kinetic curves for each sample was calculated using MARS data analysis software (BMG labtech) as in RFU/30 min. The difference between the signals obtained in the presence of the specific SSAO inhibitor (1 μM Mofegiline low signal control) and in the absence of the inhibitor (high signal control) was considered to be the specific SSAO activity in the sample. For animal tissues, the signal obtained from the non-treated group was considered to be 100% and all other groups were adjusted accordingly to yield a % response graph. IC_50_ values were calculated from the concentration-response curves using Studies software (Dotmatics, UK).

Purified recombinant human VAP-1/SSAO (rhSSAO) was obtained from CSIRO (Australia).

### Other fluorometric enzyme activity assays

MAO-A, MAO-B, DAO, LOXL2 and LOX enzymatic assays followed the same protocol as for SSAO, with slight modifications.

For recombinant human monoamine oxidases A and B (rhMAO-A and rhMAO-B, Sigma-Aldrich) the following substrates were used: 200 μM tyramine (Sigma-Aldrich) for rhMAO-A, and 100 μM benzylamine for rhMAO-B. The low controls for rhMAO-A and rhMAO-B assay were 1.5 μM clorgiline (Sigma-Aldrich) and 1 μM mofegiline, respectively.

Recombinant human diamine oxidase (rhDAO, kindly provided by Prof. Mitchell Guss, University of Sydney, Australia), inhibition assay utilized 200 μM putrescine and 10 μM of inhibitor aminoguanidine (both from Sigma-Aldrich) as substrate and low control, respectively.

rhLOXL2 (lysyl oxidase like 2) was purchased from R&D systems and assay was performed in 1.2 M urea, 50 mM sodium borate buffer, pH 8.2. The substrate used was 10 mM putrescine and 100 μM of β-aminopropionitrile (βAPN, Sigma-Aldrich) was added to the low controls.

Bovine Lysyl Oxidase (LOX) was extracted by adapting the methodology from Rucker et al. [26] from calf aorta: the homogenized tissue was washed extensively from readily soluble proteins and salts with PBS, and then extracted in 4 M urea, 50 mM sodium borate buffer, pH 8.2. The supernatant underwent buffer exchange and was concentrated by means of Amicon 10 kDa centrifuge filters (Millipore) in 1.2 M urea, 50 mM sodium borate buffer, pH 8.2 and in the presence of protease inhibitors (0.5 mM phenylmethylsulfonyl fluoride, 0.5 mM N-ethlylmaleimide, 0.5 mM p-aminobenzoic acid, 20 mg/mL soybean trypsin inhibitor, all from Sigma-Aldrich). LOX enzyme mixture was pre-treated with 0.5 mM pargyline and 1 μM mofegiline (to inhibit any potential endogenous monoamine oxidase A and B as well as SSAO), in 1.2 M Urea buffer, 50 mM sodium borate, pH 8.2. The substrate used was 10 mM putrescine and 100 μM of β-aminopropionitrile (βAPN, Sigma-Aldrich) was added to the low control. Reaction mix was made up in 1.2 M urea, 50 mM sodium borate buffer, pH 8.2.

### Off-target activity

PXS-4728A, was tested at Ricerca Inc in the “lead seeker selectivity panel”, which tests over 100 different targets.

### Pharmacokinetics and pharmacodynamics

Studies were performed by ICP Firefly, Pharmalegacy and Sundia with local ethics approval. Wistar rats were administered PXS-4728A orally at 6 mg.kg^−1^ or intravenously at 3 mg.kg^−1^, while BALB/c mice received PXS-4728A orally at 10 mg.kg^−1^ or intravenously at 5 mg.kg^−1^. Plasma was analyzed for PXS-4728A by high-performance liquid chromatography-mass spectrometry/mass spectrometry. Basic pharmacokinetic parameters were estimated using PK Solutions software (Summit Research Services).

To measure SSAO activity, animals were euthanized after 24 hours and abdominal fat as well as lung was collected. Tissue samples were surgically removed and weighed, homogenized in ice-cold HES buffer (20 mM HEPES, 1 mM EDTA, Sucrose 250 mM, 1× proteases and phosphatases inhibitor, pH 7.4) at a final concentration of 1 g/20 mL (mouse fat), 1 g/5 ml (rat lung and fat). The homogenate underwent 2000 rpm centrifugation for 5 min at 4°C and the supernatant was diluted in assay buffer (0.1 M sodium phosphate buffer), 1:20 (rat tissues) or 1:5 (mouse tissues). Supernatant was analysed using the fluorometric method for SSAO activity as described above.

### Induction of cremaster inflammation

Study was performed following UFMG (Federal University of Minas Gerais) local ethics committee approval. BALB/c mice were anesthetized and 500 ng of murine rCXCL1 in 0.2 ml of saline was administered locally by s.c. injection beneath the right scrotal skin using a 30-G needle, 2 hours before exteriorization. The left cremaster was then prepared for intravital microscopy, as previously described [27]. Briefly, an incision was made in the scrotal skin to expose the left cremaster muscle, which was then carefully removed from the associated fascia. A lengthwise incision was made on the ventral surface of the cremaster muscle using a cautery. The testicle and the epididymis were separated from the underlying muscle and were moved into the abdominal cavity. The muscle was then spread out over an optically clear viewing pedestal and was secured along the edges with a 4–0 suture. The exposed tissue was superfused with warm bicarbonate-buffered saline (pH 7.4). An intravital microscope (Olympus BX50F4) with a 20× objective lens and a 10× eyepiece was used to examine the cremasteric microcirculation. A video camera (CDD; Sony) was used to project the images onto a monitor, and the images were recorded for playback analysis using a conventional DVD. Single, unbranched cremasteric venules (25–40 μm in diameter) were selected and, to minimize variability, the same section of cremasteric venule was observed throughout the experiment. The number of rolling and adherent leukocytes was determined offline during video playback analysis. Rolling leukocytes were defined as those cells moving at a velocity less than that of erythrocytes within a given vessel. The flux of rolling cells was measured as the number of rolling cells passing by a given point in the venule per minute. A leukocyte was considered to be adherent if it remained stationary for at least 30 s, and total leukocyte adhesion was quantified as the number of adherent cells within a 100 μm length of venule. PXS-4728A (6 mg.kg^−1^) was given orally 1 hour prior to 500 ng of murine rCXCL1.

### Lipopolysaccharide (LPS) airway inflammation

6 hour time point: the study was performed by Washington Biotechnology with approval from local ethics committee. BALB/c animals were anesthetized, a midline incision was made in the neck, the muscle layers separated by blunt dissection, and 6 μg/LPS from *E. coli* strain 055:B5 injected into the trachea. The incision was closed with wound clips and the mice returned to cages. PXS-4728A (0.2 or 2 mg.kg^−1^) or dexamethasone (10 mg.kg^−1^) was given orally 1 hour prior to stimulus surgery. Dose of dexamethasone was chosen based on the inhibitory results depicted in the literature [28].

24 hour time point: the study was performed by Pharmalegacy with approval from local ethics committee. Swiss mice were anesthetized and 500 μg of LPS from *E. coli* strain 0111:B4 was intranasally instilled.PXS-4728A (4 mg.kg^−1^) or dexamethasone (10 mg.kg^−1^) was given orally 1 hour prior to stimulus. PXS-4728A (6 mg.kg^−1^) was administered a second time 6 hours post stimulus. Given the short half-life of PXS-4728, a second dose was administered to ensure that newly synthesized SSAO would also be blocked.

At 6 hours or 24 hours, mice were euthanized and bronchoalveolar lavage (BAL) was performed for recovery of airway luminal cells. Briefly, lungs were gently lavaged via tracheal cannula with 0.5 mL of PBS. The procedure was repeated twice with 0.5 mL PBS. BAL was centrifuged at 4°C with 300 g × 5 min and cells were suspended by 0.3 mL PBS. Total cell number and differential cell counts in BALF by haemocytometer. Differential cell counts (lymphocytes, eosinophils, macrophages and neutrophils) were made from cytocentrifuged preparations using cytospins and after staining with Wright-Giemsa.

### Klebsiella infection

Study was performed at the Pre-Clinical Services group at the University of North Texas Health Science Center with approval from local ethics committee. BALB/c mice were anesthetized and inoculated intranasally with 10^5^ – 10^7^ CFU of *Klebsiella pneumoniae* (UNT024-1 ATCC43816).

At 24 hours BAL was collected as described for the LPS model, following the BAL, lungs were homogenized in sterile PBS using a Polytron tissue homogenizer, serially diluted (8 × 10-fold dilutions) and all dilutions plated on Trypticase soy agar + charcoal (to prevent the effects any compound carryover) for the determination of tissue associated bacterial CFU counts. PXS-4728A (6 mg.kg^−1^) or dexamethasone (10 mg.kg^−1^) was given orally 1 hour prior to infection. For survival analysis, animals were treated 1 hour prior to infection and daily thereafter. To maintain consistency in dexamethasone treatment between models, a 10 mg.kg^−1^ dose was utilized.

### Induction of the cecal ligation and puncture insult

Study was performed following UFMG (Federal University of Minas Gerais) local ethics committee approval. The CLP procedure involved a laparotomy and ligation of the cecum, distal to the ileo-cecal valve. C57/BL6 mice were anesthetized and the cecum was punctured, with a 21-gauge needle to induce moderate sepsis, following the needle a small amount of fecal matter was extruded from each puncture. Following ligation and puncture, the cecum was returned to the abdomen, the peritoneal wall and skin incisions were closed, and the animals were allowed to recover. Sham animals received a laparotomy without manipulation of the cecum. Animals were euthanized after 6 hours following induction of sepsis and BAL as well as peritoneal cavity wash (with 1.5 ml of PBS containing 1 mM EDTA) was performed for analysis of total and differential cell counts. Lungs were removed for myeloperoxidase activity measurement as previously described [29]. Briefly, before lung removal, the pulmonary vasculature was perfused with 3 ml of PBS through the right ventricle, and the organ was removed and frozen. Upon thawing, the right lobe (0.1 g of tissue per 1.9 ml of buffer) was homogenized in a pH 4.7 buffer (0.1 M NaCl, 0.02 M Na_2_PO_4_, 0.015 M Na_2_EDTA), centrifuged at 3000 g for 10 minutes, and the pellet subjected to hypotonic lysis (1.5 ml of 0.2% NaCl solution) followed by an addition 30 seconds later of an equal volume of a solution containing NaCl 1.6% and glucose 5%. After a further centrifugation, the pellet was resuspended in 0.05 M Na_2_PO_4_ buffer (pH 5.4) containing 0.5% hexadecyl-trimethylammonium bromide (HTAB) and re-homogenized. One-milliliter aliquots of the suspension were transferred into 1.5-ml micro- tubes followed by three freeze-thaw cycles using liquid nitrogen. The aliquots were then centrifuged for 15 minutes at 3000 g to perform the assay. PXS-4728A (6 mg.kg^−1^) or dexamethasone (5 mg.kg^−1^) was given orally 1 hour prior to surgery. For survival animals were treated 1 hour prior to infection and daily thereafter. Dose for dexamethasone was chosen based on previously observed findings (unpublished results).

### House dust mite/Rhinovirus model

Study performed at the University of Newcastle under local ethics committee. BALB/c mice were sensitized and challenged intranasally to 50 μg of crude HDM extract (*Dermatophagoides pteronyssinus* extract was obtained from Greer Laboratories) on days 0, 1 and 2 followed by four exposures of 5 μg HDM daily from day 14 to day 17 delivered in 50 μl of sterile saline. Animals were then infected on day 18 with infective Human Rhinovirus 1b RV1B41 (2.5 × 10^6^ median tissue culture infective dose) intranasally [30]. Mice were intranasally administered 50 μl infective or ultraviolet light (UV)–inactivated RV1B41. Mice were euthanized 24 hours after the rhinovirus challenge. BAL analysis was performed for total and differential counts and airway hyperreactivity (AHR) was assessed invasively by measurement of total lung resistance and dynamic compliance (BUXCO) in response to methacholine challenge as previously described [31]. PXS-4728A (2 mg.kg^−1^), dexamethasone (2 mg.kg^−1^), azithromycin (25 mg.kg^−1^) or saline was delivered (ip.), post final intransal HDM challenge and prior to intranasal Rhinovirus inoculation. Dose of azithromycin was chosen based on previously described findings, in respiratory viral infection models [32].

### Survival analysis

Experiments complied with stringent ethics protocols from UNTHSC (for the Klebisella model) or UFMG (for the CLP model). An important aspect of the ethics regulations was that pain and distress be minimized whenever possible. Therefore, experiments were performed as to avoid animal discomfort, unless necessary to achieve the goals of the study. Animals were closely monitored and if animals reached >20% weight loss or were in moribund state they were euthanized. Once animals started to display clinical signs of illness, investigators monitored the subjects more frequently to ensure timely identification of moribund animals. Investigators were obligated to make every effort to identify and humanely euthanize moribund animals that had not responded to treatment.

### Statistical analysis

Statistical analysis was performed by one-way ANOVA with Newman-Keuls Multiple Comparison Test, unless otherwise stated. Data are represented as Mean with S.E.M.

## Results

### PXS-4728A compound profile

PXS-4728A exhibited an IC_50_ of <10 nM against the human VAP-1/SSAO enzyme and this activity was maintained across all the mammalian species tested (Table 1). The compound was found to be more than 500-fold selective for VAP-1/SSAO over all the related human amine oxidases. PXS-4728A shares the excellent potency, selectivity (as seen in Table 1) and oral bioavailability (Table 2) with the previously described compound PXS-4681A [24] but has a more balanced blood/plasma ratio (1.2 for human and dog, 1.45 for rat) due to substitution of the sulfonamide group with an amide, removing all activity for carbonic anhydrase II and thereby avoiding the potential for sequestration by erythrocytes in whole blood. Like PXS-4681A, PXS-4728A is stable in human, rat and dog plasma (>90% remaining after 1 hour incubation) and has high water solubility >10 mg.ml^−1^ at pH 7.4. PXS-4728A is a mechanism-based inhibitor of VAP-1/SSAO with Ki of 175 nM and Kinact of 0.68 min^−1^ when determined by time-dependent inhibition using Kitz-Wilson plot.

**Table 1:** **Activity and selectivity of PXS-4728A** ***in vitro***

**Assay**	**IC**_**50**_	
Recombinant human VAP-1/SSAO (AOC3)	5 nM	
Gonadal fat tissue homogenate; mouse, rat, dog, rabbit	<10 nM	
**Assay**	**IC**_**50**_	**Selectivity**
Recombinant diamine oxidase (AOC1)	12.0 μM	>2000x
Recombinant retina specific amine oxidase (AOC2)	2.76 μM	>500x
Purified lysyl oxidase from lung fibroblasts	13.2 μM	>2000x
Recombinant lysyl oxidase like 2	10.4 μM	>2000x
Recombinant monoamine oxidase A	>100 μM	>2000x
Recombinant monoamine oxidase B	2.7 μM	>500x
Recombinant lysine demethylase	>10 μM	>2000x

**Table 2:** **Pharmacokinetic profile of PXS-4728A in rodents**

	**Rat**	**Mouse**
**Oral dose**	**6 mg.kg** ^**−1**^	**10 mg.kg** ^**−1**^
Bioavailability (%)	55	92
Apparent concentration at curve maximum C_max_ (μg.ml^−1^)	0.72	1.38
Time of maximal concentration T_max_ (hour)	0.83	0.25
Clearance CL (ml.kg.min^−1^)	45	98
**Intravenous dose**	**3 mg.kg** ^**−1**^	**5 mg.kg** ^**−1**^
Apparent concentration at curve maximum C_max_ (μg.ml^−1^)	4.21	1.6
Time of maximal concentration T_max_ (min)	0.03	0.03
Clearance CL (ml.kg.min^−1^)	23	80
Apparent half-life, t_1/2_ (h)	1.1	0.6

### Pharmacokinetic parameters

The pharmacokinetic (PK) properties of PXS-4728A were investigated in rats and mice (n = 3 in each group) and are reported in Table 2. In rats when dosed at 6/3 mg.kg^−1^ (oral/i.v.), PXS-4728A presented a bioavailability of >55% and a half-life of approximately 1 hour (i.v.). As the models of inflammation in this study were performed in mice, the PK profile of PXS-4728A was also evaluated in BALB/c mice (Table 2). Mice were dosed at 10/5 mg.kg^−1^ (oral/i.v.) and PXS-4728A presented a high bioavailability of >90%, a maximal concentration of 1.38 μg.ml^−1^ was reached and all other derived PK values followed the same trend as the rat data.

PXS-4728A does not have any significant off-target activity when tested against other amine oxidases or over 100 different macromolecular targets. PXS-4728A does not induce cell toxicity or phospholipidosis in HepG2 cells (highest concentration tested 100 μM).

### Pharmacodynamic (PD) profile

To determine the inhibition of SSAO by PXS-4728A, abdominal adipose tissue was collected at 24 hours after dosing. The adipose tissue was chosen as it had been previously described to contain high quantities of SSAO, allowing a large assay window to detect enzyme inhibition [33].

**Figure 2:**
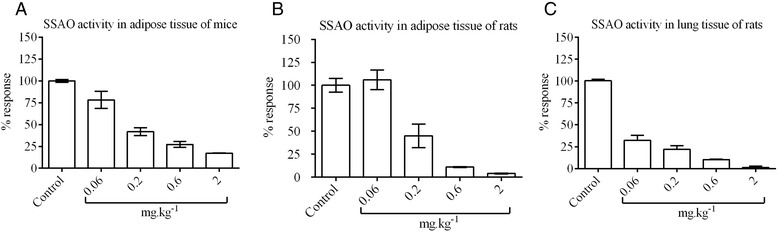
**Pharmacodynamic profile of PXS-4728A 24 hours after dosing.** Measurement of total VAP-1/SSAO activity (%) compared to untreated control; **(A)** single time point with escalating doses in the **(A)** adipose tissue of BALB/c mice **(B)** adipose tissue of Wistar rats **(C)** lung of Wistar rats.

Mice administered orally with a single dose of 0.2 and 0.6 mg.kg^−1^ showed a 60-70% reduction in SSAO activity compared to the control group (slope of ~153 in a 1% tissue/buffer solution) at 24 hours, whilst 2 mg.kg^−1^ dosing resulted in over 80% inhibition of the enzyme (Figure 2A). Due to the focus of the present study on respiratory disease models, activity of the enzyme was then tested in the mouse lung; however this procedure was unsuccessful as the AR/HRP assay available did not provide enough sensitivity. To overcome this, the pharmacodynamic profile was analyzed in the rat.

Rats administered with an oral dose of 0.2 mg.kg^−1^ showed a 55% reduction in SSAO activity in the adipose tissue compared to the control group (slope of ~116 in a 1% tissue/buffer solution), whilst 0.6 and 2 mg.kg^−1^ dosing resulted in over 80% inhibition of the enzyme (Figure 2B). SSAO activity in the lungs of rats showed that 0.6 and 2 mg.kg^−1^ resulted in near-total inhibition of the enzyme at 24 hours (Figure 2C) compared to the control group (slope of ~138 in a 1% tissue/buffer solution).

Given the comparable PD results in the adipose tissue between the mouse and rat it was presumed that the PD in the lung would be similar (SSAO enzyme should be inhibited).

The results from mouse PK and PD indicate that doses of 2 mg.kg^−1^ or greater were needed to achieve maximal inhibition of SSAO function. Since the anti-inflammatory effects are seen only when complete inhibition of the enzyme activity is achieved [24] the models described in this study are focused on doses of ≥ 2 mg.kg^−1^.

### Inhibition of cell adhesion and rolling

**Figure 3:**
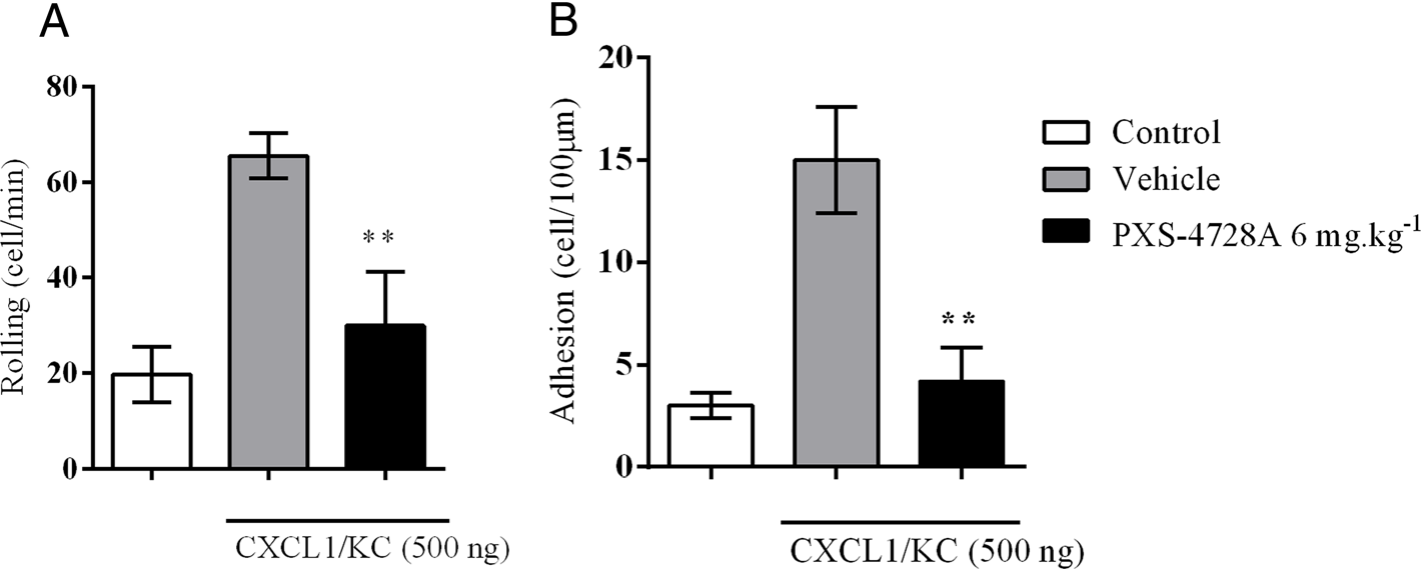
**Inhibition of cell adhesion and rolling by PXS-4728A.** Inhibition of VAP-1/SSAO dampened the **(A)** rolling and **(B)** adhesion of leukocytes after CXCL1 stimulation. N = 5-6 per group; **p < 0.01 over vehicle.

At the cellular level VAP-1/SSAO knockout animals have increased leukocyte velocity, causing decreased adhesion in the cremaster model [34]. However, the direct effects of VAP-1/SSAO small molecule inhibitors on neutrophil egress from the microvasculature *in vivo* have not been determined. The mouse cremaster model allows visualization of the early steps of PMN (polymorphonuclear leukocyte) migration. After exposure of the cremaster muscle, cell recruitment was induced by cytokine injection and analyzed by an intravital microscopy. CXCL1/KC caused PMN to tether and strongly adhere to endothelia cells and subsequently migrate. Transendothelial migration is difficult to measure in a two dimensional space therefore the quantitative analysis was restricted to rolling and adhering leukocytes (Figure 3). CXCL1/KC increased the number of rolling and adhering leukocytes and this was abolished by a 6 mg.kg^−1^ oral dose of PXS-4728A (Figure 3A/B). These data demonstrate that the enzymatic activity of VAP-1/SSAO is required in the initial steps of neutrophil migration and inhibition of VAP-1/SSAO could potentially prevent the events that lead to the migration of leukocytes to the site of inflammation.

### VAP-1/SSAO inhibition of neutrophil migration

**Figure 4:**
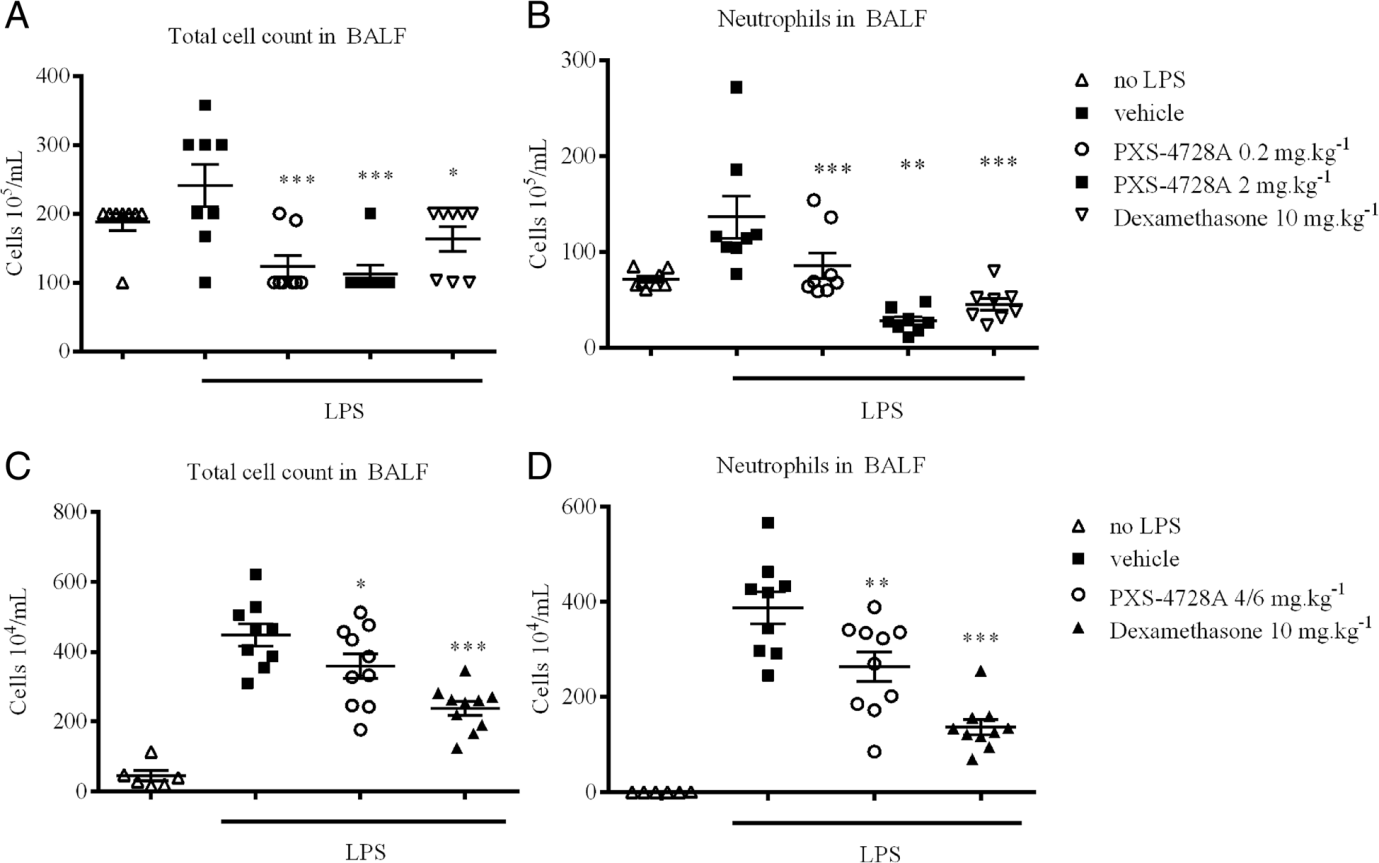
**Inhibition of LPS induced inflammation.** LPS caused an increase in total number of cells and neutrophils in the BALF, as measured 6 and 24 hours after the insult. Pre-treatment with PXS-4728A (VAP-1/SSAO inhibitor) concentration-dependently reduced the influx of **(A)** total cells and **(B)** neutrophils at 6 hours. Pre-treatment with 4 mg/kg of PXS-4728A (VAP-1/SSAO inhibitor), followed by a repeat treatment of 6 mg/kg at 6 hours reduced the influx of **(C)** total cells and **(D)** neutrophils. N = 6-12 per group; *p < 0.05, **p < 0.01, ***p < 0.001 over vehicle.

To demonstrate that inhibition of leukocyte rolling and adhering by PXS-4728A translates into a reduction in inflammation; a model of neutrophilic acute lung injury driven by Lipopolysaccharide (LPS) was employed. LPS is a component of the cell wall of Gram-negative bacteria and is a well-established stimulant of neutrophilic inflammation. Animals were exposed to LPS and neutrophil recruitment into the lungs was monitored at different time points. As expected, LPS increased the number of airway neutrophils in bronchoalveolar lavage fluid (BALF) at 6 hours (Figure 4A/B) in BALB/c mice. Neutrophil inflammation was highly dependent on VAP-1/SSAO enzymatic function, as PXS-4728A dampened migration significantly and comparable of the reduction by dexamethasone.

To strengthen this finding a different mouse strain, SWISS, at the 24 hour time time point was used. At twenty-four hours post LPS stimulation, airway inflammation was present as can be seen by the increase in total cell counts and neutrophils (Figure 4C/D). PXS-4728A applied 1 hour before and 6 hours after the insult still reduced inflammation. This demonstrates that independent of the mouse strain PXS-4728A can diminish inflammation during early and late responses.

**Figure 5:**
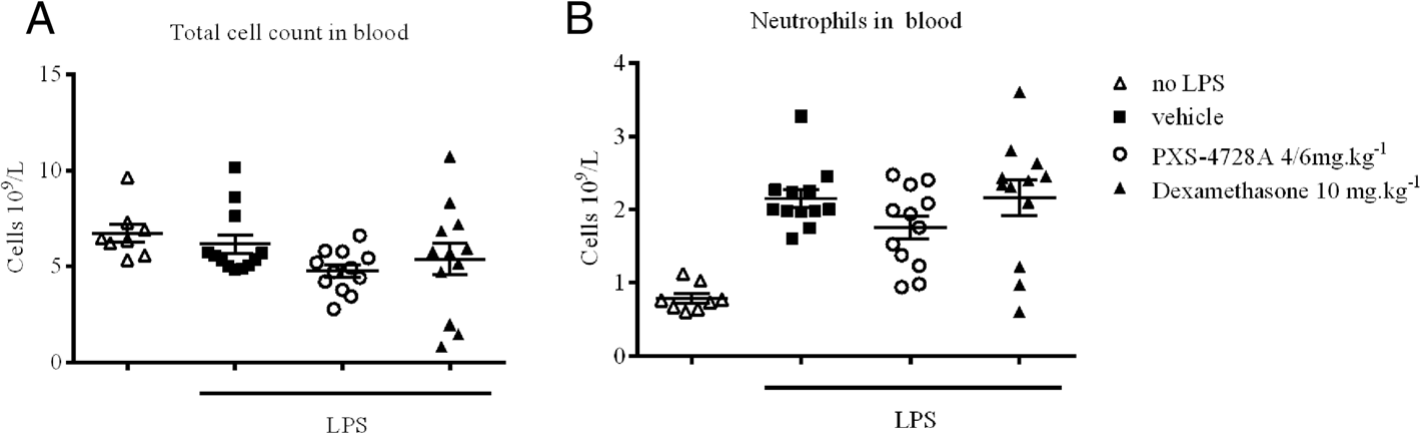
**Circulating leukocytes during LPS induced inflammation.** LPS caused increased neutrophilia in the blood vessel as measured 24 hours after the insult. Pre-treatment with PXS-4728A (VAP-1/SSAO inhibitor) had no effect on **(A)** total cells or **(B)** circulating neutrophils. N = 8 in the non LPS group, N = 12 in the LPS treated groups.

It is plausible that limiting neutrophil migration into the airway spaces could lead to a build-up of cells in the blood vessels and therefore blood counts were performed. There was an increase in neutrophils in the circulation upon LPS stimulus and this increase was unaffected upon VAP-1/SSAO inhibition (Figure 5A/B). These results confirm that reducing emigration of cells from the blood vessel (via VAP-1/SSAO) has no effect on the accumulation of blood circulating neutrophils.

**Figure 6:**
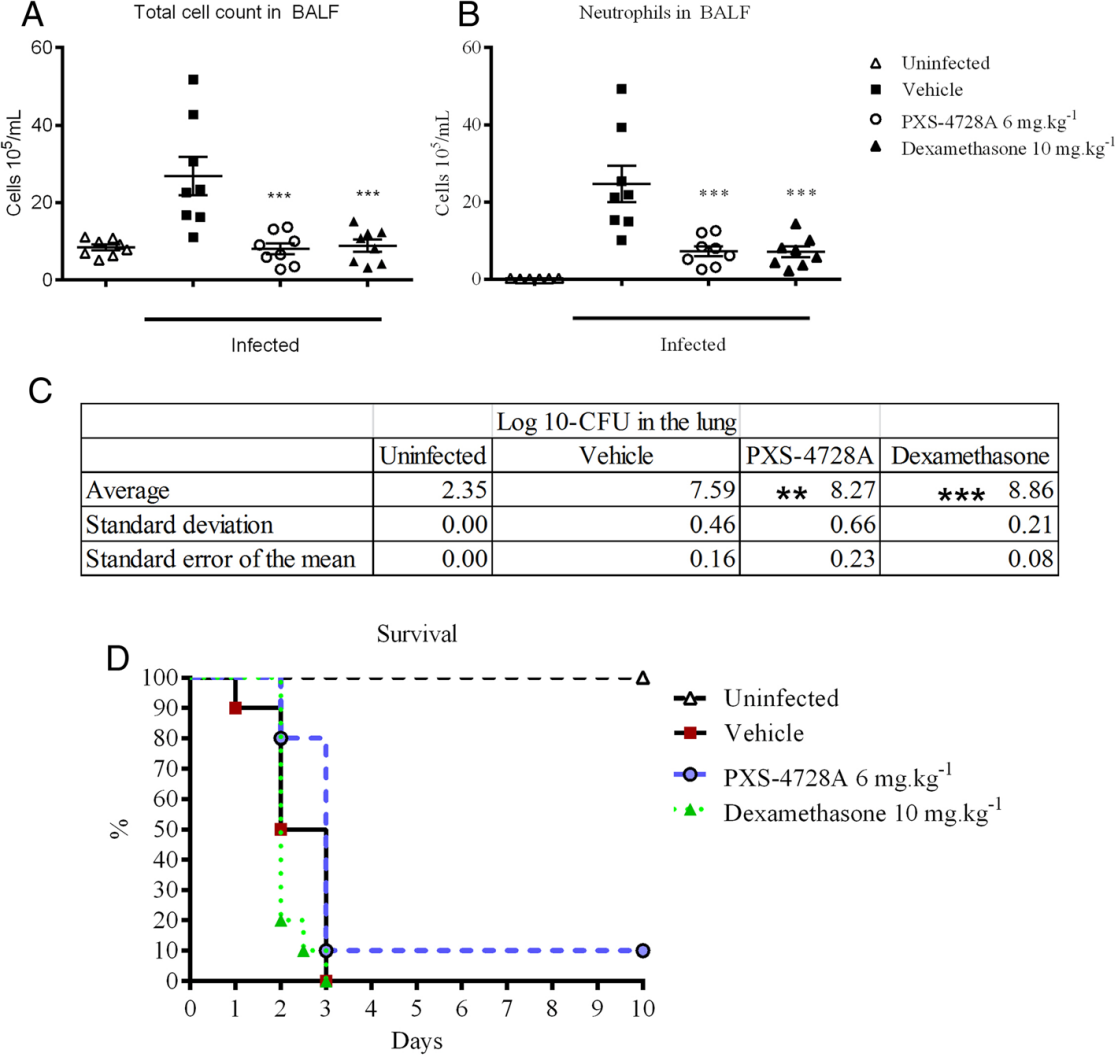
**Inhibition of inflammation during** ***Klebsiella pneumonia*** **infection.** Mice were inoculated intranasally with 0.05 mL of ~10^5^ – 10^7^ input CFU of *Klebsiella pneumoniae*. Inhibition of VAP-1/SSAO dampened **(A)** total leukocyte as well as **(B)** neutrophil migration into the airway spaces and increased bacterial load **(C)**. **(D)** Although bacterial counts were higher in the VAP-1/SSAO inhibited group, survival was not affected;comparison in the survival of dexamethasone treated animals to PXS-4728A was of p < 0.0018. N = 8 per group for the BALF and bacterial counts, N = 10 for survival; **p < 0.01, ***p < 0.001 over vehicle.

Bacterial infections are often the underlying cause of chronic lung inflammation, to further investigate the potential beneficial effects of SSAO/VAP-1 inhibition in lung inflammation, animals underwent a bacterial infection model. Animals were infected with *Klebsiella pneumoniae* as this is a frequently occurring Gram-negative bacteria with high replicating properties. Animals inoculated intranasally with *Klebsiella pneumoniae* showed an increase in airway leukocytes, particularly neutrophils (Figure 6A/B). Upon inhibition of VAP-1/SSAO, the migration of these cells to the site of infection was significantly dampened, similar to the effects of dexamethasone. It is important to note that although VAP-1/SSAO inhibition lead to an increase in bacterial compared to the vehicle control (Figure 6C), this did not reach a log unit difference *[vehicle (7.59 LOG 10-CFU)* and *PXS-4728A (8.27 LOG 10-CFU)*], this is of particular importance as bacteria replicate exponentially, in addition the survival of animals was not compromised (Figure 6D). It is noteworthy that when inflammation was ablated by the use of glucocorticosteroids, the difference in survival was significant between the two treatment groups. This demonstrates that inhibition of cell adhesion/rolling via VAP-1/SSAO reduces rather than abolishes neutrophil infiltration, allowing a sufficient neutrophilic response with which to combat an infection.

**Figure 7:**
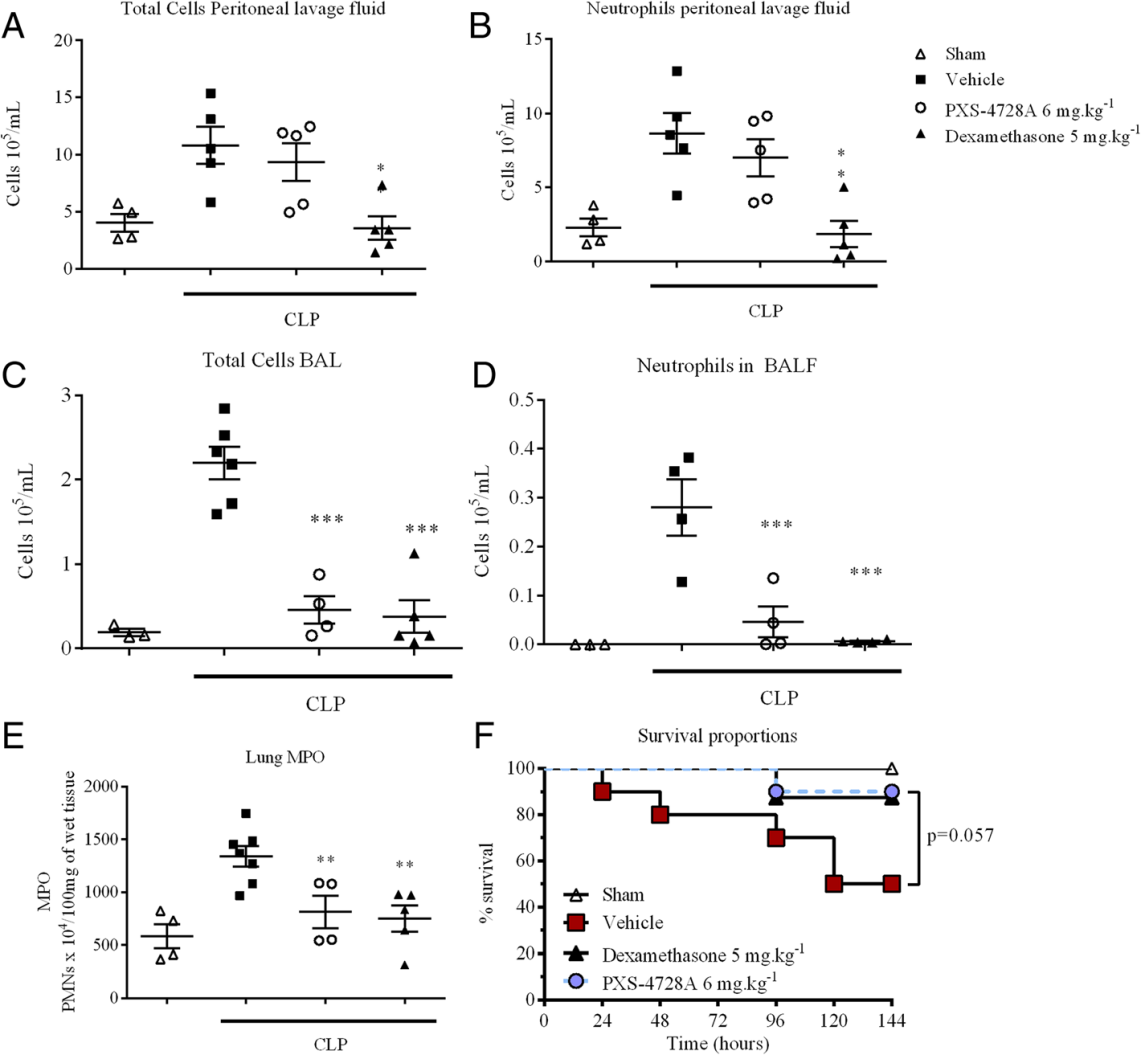
**Inhibition of inflammation during CLP induces sepsis.** Cecal ligation and puncture (CLP) was induced by ligating and perforating the cecum. Cecal contents were expressed through the perforation. The cecum was placed back into the peritoneal cavity and the peritoneal wall and skin incisions were then closed. 6 hours after surgery **(A)** total cells **(B)** and neutrophils were analysed in the peritoneal lavage. Inhibition of inflammation by VAP-1/SSAO regulation was seen in the bronchoalveolar lavage, by a decrease in **(C)** total cell counts, **(D)** neutrophils and **(E)** MPO. Treatment with PXS-4728A increased survival **(F)**. N = 4-6 per group for the BALF, peritoneal lavage and MPO; N = 8/10 per group for survival; **p < 0.01, ***p < 0.001 over sham.

The results described above show that after a localised stimulus by LPS and *Klebsiella pneumoniae* infection, neutrophil extravasation into the airways was VAP-1/SSAO dependent. To further expand the role of SSAO/VAP-1 in respiratory inflammation, neutrophil migration during a generalized systemic infection was studied. Sepsis is a condition characterized by dysregulated systemic inflammatory response. Sepsis induced by cecal ligation and puncture (CLP) is the most frequently used model as it has the advantage of closely resembling the progression and characteristics of human sepsis [35]. CLP is triggered by a polymicrobial infection, which begins as peritonitis and is followed by a systemic inflammatory response with a destructive lung inflammatory component (acute lung injury). As such, this model has a very robust neutrophilic element in the peritoneal and lung compartments as well as an aggressive systemic bacterial infection. The CLP model was performed in mice pre-treated with the VAP-1/SSAO inhibitor. The proportion of total peritoneal inflammatory cells as well as peritoneal neutrophils at 6 hours (Figure 7A/B) was not affected by VAP-1/SSAO inhibition but was greatly diminished by dexamethasone indicating that the defense system during a severe infection is not compromised.

Interestingly, VAP-1/SSAO inhibition significantly dampened the lung injury induced by CLP, with a reduction in total cells, neutrophils, as well as myeloperoxidase in the lung (Figure 7C-E). Although it did not reach significance, survival benefits were evident after VAP-1/SSAO inhibition (Figure 7F), as PXS-4728A administration increased survival rates compared with control animals from 50% to 90% (p = 0.057). These results show that VAP-1/SSAO has distinct roles in different tissues, with inhibition of inflammation in the lung, yet no effect on leukocytes in the peritoneal cavity during very severe infection. This highlights a beneficial effect of inhibition of VAP-1/SSAO as it is able to reduce lung injury but does not reduce systemic host defence.

**Figure 8:**
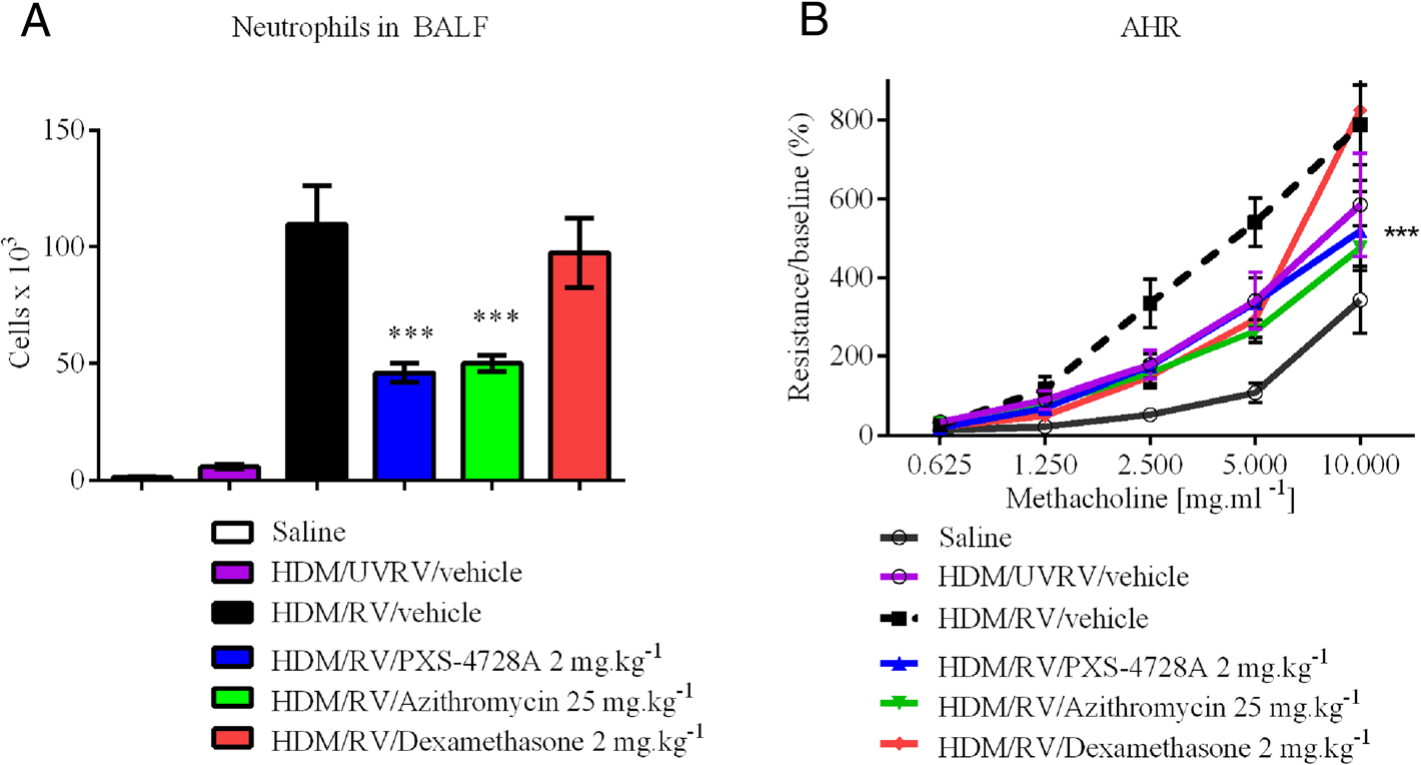
**Inhibition of inflammation during viral induced asthma exacerbation.** Allergic airways disease was established with house dust mite (HDM) sensitization followed by HDM challenge. RV1b infection was superimposed on the allergic mice to induce an exacerbation and results analysed 24 hours post infection. PXS-4728A reduced **(A)** total BALF neutrophils and **(B)** significantly reduced airways hyperreactivity to methacholine challenge in ventilated mice as of 2.5 mg.ml^−1^. N = 8 per group for AHR and N = 4 per group for BALF; ***p < 0.001 over HDM/vehicle/RV. AHR analysed by two-way ANOVA with Newman-Keuls Multiple Comparison Test.

Having established that selective VAP-1/SSAO inhibition alters the migration of neutrophils in response to acute local and systemic bacterial lung inflammation whilst still allowing normal neutrophil defense; the role of VAP-1/SSAO during a viral infection was assessed. Rhinovirus infection was performed concomitant with the house dust mite asthma model in order to mimic a viral exacerbated asthmatic response. Rhinoviruses (RV) have been identified as being the main perpetrators of asthma exacerbations, which is characterized by increases in airway hyperreactivity and neutrophilia [31]. Inhibiting VAP-1/SSAO prior to the viral infection significantly dampened the neutrophil infiltrate in a manner comparable to that of the macrolide azithromycin (Figure 8A). In this model azithromycin - a macrolide which shows great promise in the clinic for the treatment of asthma- was used as a comparator as dexamethasone has no impact on RV1b induced neutrophilic inflammation of the HDM allergic lungs. The reduced cellular infiltrate following VAP-1/SSAO inhibition correlated with a significant reduction to the exacerbation of airways hyperreactivity (AHR) in response to methacholine challenge (Figure 8B).

## Discussion

These studies clearly show that inhibition of the enzymatic activity of VAP-1/SSAO by PXS-4728A diminishes lung inflammation in a variety of models suggesting that this mechanism plays an important role in respiratory diseases. PXS-4728A is a highly selective inhibitor of VAP-1/SSAO, with exquisite pharmacodynamic and pharmacokinetic properties, that inhibits neutrophil rolling and tethering in the mouse cremaster model. As a consequence, lung inflammation was reduced by PXS-4728A during LPS stimulus, *Klebsiella pneumoniae* infection and CLP injury without impacting the survival or susceptibility to infection, in addition to improving outcomes of RV induced asthma. It is important to highlight that PXS-4728A diminished, but did not abolish neutrophil influx to the lung and did not change neutrophil counts in circulation, affording a more beneficial approach to lung inflammation than CXCR2 blockers, that have been shown to cause neutropenia [19].

VAP-1/SSAO is expressed on the surface of endothelial cells and has its enzymatically active domain outside of the cell membrane. VAP-1 was originally discovered to be an adhesion molecule [36], however cloning of the antigen revealed that it also had semicarbazide-sensitive amine oxidases (SSAO) activity [37]. Following these findings, a number of *in vitro* studies demonstrated that anti-VAP-1 mAbs as well as small molecule SSAO inhibitors blocked leukocyte-endothelial cell interactions [38,39], suggesting that both the adhesion and enzymatic functions converge to a common process which regulates leukocyte recruitment *in vitro*.

Deletion of VAP-1/SSAO increased leukocyte velocity, thereby causing a decrease in leukocyte adhesion in the mouse cremaster model, with similar results obtained using an anti-mouse VAP-1 monoclonal antibody [34]. VAP-1/SSAO potentially acts as an important breaking mechanism by reducing the velocity of rolling cells allowing leukocytes to survey the endothelial cells for signs that are required for subsequent adhesion [40]. As such, it was not surprising to find that the inhibition of the enzymatic activity by PXS-4728A in the cremaster model impaired the capacity of leukocytes to interact with the vascular endothelium. However, for the first time, direct pharmacological *in vivo* evidence is presented that VAP-1/SSAO inhibitor affects the rolling of neutrophils and in fact acts as a breaking mechanism by decreasing leukocyte-endothelial interaction.

The exact mechanisms of SSAO-leukocyte binding were not elucidated here. However, a number of studies have suggested that SSAO binds to arginine residues present on the granulocyte Siglec-10 receptor [41]. Given that SSAO also binds to lysine residues [42,43], it is possible that lysine residues present on the neutrophil surface may facilitate the targeting of endothelial bound SSAO, which could assist neutrophil tethering and rolling on the endothelium. In fact, a number of neutrophil-bound receptors have been found to be rich in lysine residues such as CD44 as well CD68 [44-47], making these receptors potential ligands for SSAO.

The functional importance of cell adhesion molecules on leukocyte behaviour has been predominantly analysed in the systemic microcirculation, with ICAM-1 and various selectins playing a key role on leukocyte adhesion and rolling [5,11]. In the current study it was demonstrated that VAP-1/SSAO is important in mediating adhesion and rolling in the systemic circulation, as seen by inhibition of these steps by PXS-4728A in the cremaster model. However, the adherence between leukocytes and endothelial cells in injured venules of the systemic circulation is somewhat different to that of the pulmonary microcirculation with 90% of neutrophils originating from the capillaries [48], where adhesion has not been implicated [14]. In order for leukocyte migration to occur in the lung capillaries, leukocytes must undergo deformation [16] and the rolling is dependent on ICAM-1 and E-selectin [14,16]. Although the current study did not visualize the effects of VAP-1/SSAO on adhesion and rolling in lung microcirculation it is clear that SSAO is involved in those processes, as PXS-4728A inhibited neutrophil infiltration driven by different stimulus’ into the lung.

PXS-4728A reduced LPS-induced neutrophil invasion into the lung. Consistent with these data it has been shown that VAP-1/SSAO moderates leukocyte migration and, inhibition of enzyme activity prolonged survival post LPS-induced endotoxemia, whilst also reducing serum levels of TNF-α and IL-6 [49]. Subsequent reports confirmed the role of VAP-1/SSAO during LPS stimulation in the lung; with inflammatory cells in the BALF being significantly higher in the VAP-1/SSAO transgenic than in the non-transgenic mice, while blocking SSAO activity reduced the number of neutrophils as well as a number of related cytokines [24,25,50].

PXS-4728A also reduced bacterial driven neutrophil influx and lung inflammation. A number of studies have demonstrated the importance of neutrophils for the clearance of infections [5]. Indeed, patients who have severe neutropenia are highly susceptible to infection [51,52]. Therefore, it was important to study the consequences of VAP-1/SSAO inhibition on bacterial infection. The data presented thus far in the literature are somewhat contradictory; *Koskinen et al.* described that the antimicrobial immune responses were affected by the deletion of VAP-1/SSAO upon infection with either *Staphylococcus aureus* (gram positive bacteria) or *coxsackie B4* virus. However, when the function of VAP-1 was neutralized using small molecule enzyme inhibitors and anti-VAP-1 Abs, rather than by gene deletion, no significant impairment in antimicrobial response against these same infections was reported [53]. The latter had also been observed by *Stolen et al.*, in which the mortality of VAP-1/SSAO deficient animals infected with the gram-negative bacteria *Yersinia enterocolitica* was not higher than that of similarly infected WT littermates [34]. Our results are in agreement with the concept that small molecule enzyme inhibitors do not negatively impact on the survival of animals upon infection or sepsis but diminish neutrophil load in the BALF. VAP-1/SSAO inhibition led to an increase in bacterial load during *Klebsiella pneumoniae* infection that was insufficient to impart on survival of the animals. These studies, therefore, show that treatment with a VAP-1/SSAO inhibitor, is accompanied by neutrophil recruitment at a sufficient level to effectively combat infection, yet in low enough numbers to diminish lung injury. Consistent with these findings, VAP-1/SSAO inhibition significantly dampened the lung injury induced by CLP induced sepsis. Although treatment with PXS-4728A demonstrated an improved survival over untreated animals, this did not reach significance, which might be attributed to the low number of animals in the model.

It is noteworthy that the properties of PXS-4728A are specific to neutrophil extravasation and not an off-target effect. When animals were infected with *Klebsiella pneumoniae* they had no impairment in survival, suggesting no issues with neutrophil phagocytosis. At the 6 hour time point in the CLP model, SSAO activity was completely abolished (given the PK/PD profile of PXS-4728A). At this stage MPO activity returned to baseline levels, which was concomitant with a significant reduction in neutrophils. Thus indicating that inhibition of myeloperoxidase was a direct effect of the reduction in neutrophils. In addition, there was no evidence that the compound could have direct effects on bacteria, as no direct homolog with AOC3 has yet been described for bacterial enzymes. Although *Klebsiella pneumoniae* contains a related TPQ, given the high specificity of PXS-4728A for AOC3 over the AOC1 and AOC2 gene products, it can be presumed that a direct effect of PXS-4728A on the bacteria is highly unlikely*.*

The beneficial effects of inhibiting VAP-1/SSAO in bacterial inflammation models were extended to another clinically relevant setting. Asthma exacerbations are most commonly triggered by infection with viruses detected in 85% of asthmatic hospitalizations and 2/3rds of these are Rhinovirus (RV) [54,55]. Neutrophils are the key cell type in the immune response to RV infection and have been strongly implicated asthma pathogenesis with neutrophil degranulation associated with asthmatic severity [56] and neutrophilic TNF-α required for the induction of AHR in mouse models [57]. Azithromycin was used as a comparator, as macrolide therapy is showing promise in the clinic, with *Brusselle et al.* reporting that azithromycin treatment reduced exacerbation rates in patients with severe neutrophilic asthma (although significance was lost when all patients were analysed together) [58]. PXS-4728A reduced cellular infiltrate and significantly reduced airways hyperreactivity (AHR), an effect that was comparable to that of the macrolide azithromycin. Therefore, the anti-inflammatory properties of PXS-4728A can dampen Rhinovirus- induced asthma exacerbation.

It is important to point out that data from a single experimental mouse model needs to be carefully interpreted and its limitations appreciated. The use of dexamethasone as a comparator is a classic example of this limitation. It is well appreciated that in the clinical setting neutrophilic inflammation is less responsive to dexamethasone treatment, however, in acute rodent models it has been shown that dexamethasone is very effective [24,28]. In the current study dexamethasone was utilized as a broad anti-inflammatory agent as it is currently one of the gold standards for acute inflammation. By using various models of lung inflammation in different strains as well as the use of relevant comparators, dexamethasone and azithromycin, the limitation of single models should have been overcome and the data should enable the development of PXS-4728A for respiratory diseases.

## Conclusions

Taken together, these results show the anti-inflammatory effects of inhibiting VAP-1/SSAO enzymatic function and highlight the potential of PXS-4728A as a novel therapeutic tool in diseases that are characterized by a neutrophilic pattern of inflammation, including acute lung injury, chronic obstructive pulmonary disease, cystic fibrosis, and bronchiolitis obliterans syndrome.

## Abbreviations

BAL: Bronchoalveolar lavage
BALF: Bronchoalveolar lavage fluid
CFU: Colony forming unit
CLP: Cecal ligation and puncture
COPD: Chronic obstructive pulmonary disease
CXCL1/KC: Chemokine (C-X-C motif) ligand 1
DAO: Diamine oxidase
HDM: House dust mite
IC_50_: Half maximal inhibitory concentration
ICAM-1: Intercellular adhesion molecule 1
IL: Interleukin
I.P.: Interperitoneal
I.V: Intravenous
K_i_: Inhibitor constant
k_inact_: Rate of inactivation
LOX: Lysyl oxidase
LPS: Lipopolysaccharide
MAO-A: Monoamine oxidase A
MAO-B: Monoamine oxidase B
MPO: Myeloperoxidase
PMN: Polymorphonuclear leukocyte
RV: Rhinovirus
SSAO: Semicarbazide-sensitive amine oxidase
VAP-1: Vascular adhesion protein-1
